# New Hydrazones Bearing Thiazole Scaffold: Synthesis, Characterization, Antimicrobial, and Antioxidant Investigation

**DOI:** 10.3390/molecules200917325

**Published:** 2015-09-18

**Authors:** Cristina Nastasă, Brîndușa Tiperciuc, Mihaela Duma, Daniela Benedec, Ovidiu Oniga

**Affiliations:** 1 Department of Pharmaceutical Chemistry, “Iuliu Hațieganu” University of Medicine and Pharmacy 41 Victor Babeș Street, RO-400012 Cluj-Napoca, Romania; E-Mails: brandu32@yahoo.com (B.T.); onigao65@yahoo.com (O.O.); 2 State Veterinary Laboratory for Animal Health and Safety, 1 Piața Mărăști Street, 400609 Cluj-Napoca, Romania; E-Mail: duma.mihaelacj@yahoo.com; 3 Department of Pharmacognosy, “Iuliu Hațieganu” University of Medicine and Pharmacy, 12 Ion Creangă Street, RO-400010 Cluj-Napoca, Romania; E-Mail: dbenedec@umfcluj.ro

**Keywords:** thiazole, hydrazide, acyl-hydrazone, antibacterial, antifungal, antioxidant

## Abstract

New series of hydrazones **5–18** were synthesized, in good yields, by reacting 4-methyl-2-(4-(trifluoromethyl)phenyl)thiazole-5-carbohydrazide with differently substituted benzaldehyde. The resulting compounds were characterized via elemental analysis, physico-chemical and spectral data. An antimicrobial screening was done, using Gram (+), Gram (−) bacteria and one fungal strain. Tested molecules displayed moderate-to-good growth inhibition activity. 2,2-Diphenyl-1-picrylhydrazide assay was used to test the antioxidant properties of the compounds. Monohydroxy (**14–16)**, *para*-fluorine (**13**) and 2,4-dichlorine (**17**) derivatives exhibited better free-radical scavenging ability than the other investigated molecules.

## 1. Introduction

Microbial resistance has been, for more than a few decades, a threat to the effectiveness of common therapy. In the case of patients at risk, this can involve the extension of the disease or even death [1,2,3,4]. Pathogens have developed several types of resistance (natural/intrinsic and acquired) and different installation mechanisms. The most alarming resistance to conventional treatment is cited for nine bacteria: *Escherichia coli*, *Klebsiella pneumoniae*, *Staphylococcus aureus*, *Streptococcus pneumoniae*, *Salmonella* and *Shigella* species, *Neisseria gonorrhoeae*. *Candida* species infections are a public health problem worldwide, the most common form of fungal infection. Invasive forms have high rate of morbidity and mortality, especially in patients with cancer, immunocompromise, new-borns, or those in the intensive care unit. There is reported increased resistance to azoles (fluconazole) and the emergence of strains resistant to echinocandins, the newest class of antifungal agents [5].

Globally, efforts are being made for the control of microbial resistance phenomenon, of its spread, effects, treatment costs, and towards finding new therapeutic protocols. In this context of the alarming development of the microbial resistance phenomenon, the discovery of new effective substances is required urgently.

Reactive oxygen species are produced in physiological and metabolic processes of the oxidative reactions that threaten living organisms. Normally, these radical species are removed by enzymatic and non-enzymatic antioxidant mechanisms. However, in certain circumstances, the increase of proportion of oxidants and decrease of antioxidants cannot be prevented. In this case, we can speak of oxidative stress, a phenomenon involved in the onset and progression of over 100 diseases (cancer, cardiovascular diseases, neurodegenerative disorders, rheumatoid arthritis, *etc.*). Antioxidants are molecules, natural or synthetic, capable of interacting with free radicals and stopping their chain reactions before essential vital molecules are damaged [6,7,8].

Scientific literature is more and more focused on Schiff bases containing heterocyclic systems, due to their large variety of biological properties. New series of hydrazones have been synthesized and investigated for their antitumor, antioxidant [9], and antimicrobial activities [10,11,12,13].

From pentatomic heterocycles, thiazole represents a very important scaffold in medicinal chemistry [14,15]. There are many compounds bearing this fragment which are used in therapy, for treating inflammation [16], oxidative stress [17,18], bacterial infections [19,20,21], hyperglycemia, hyperlipidemia [22], cancer [23,24,25], schizophrenia, hypertension, HIV infection, hypnotics, allergy, *etc.*

In new drug development studies, a combination of different pharmacophores in the same molecule may lead to new compounds having higher biological activity. Therefore the combination of thiazole- and hydrazone-type compounds might provide new effective drugs for the treatment of multidrug resistant microbial infections and for diminishing oxidative processes. Prompted by all these relevant data, we propose here the synthesis of new hybrid molecules, which gather the two pharmacophores, and the investigation of their antimicrobial and antioxidant potential.

## 2. Results and Discussion

### 2.1. Chemistry

**Scheme 1:**
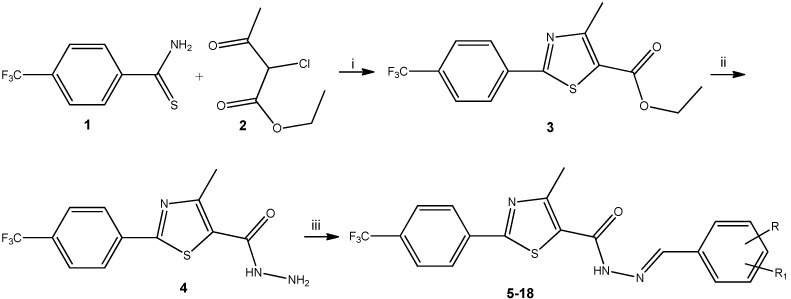
Synthesis of new acyl-hydrazones bearing thiazole scaffold. i: ethanol/reflux; ii: NH_2_NH_2_·H_2_O/ethanol/reflux; iii: substituted benzaldehyde/H_2_SO_4_/ethanol/reflux.

The reaction sequences used for the synthesis of target compounds are shown in Scheme 1. In order to obtain thiazole heterocycle, in good yield (80%), Hantzsch thiazole synthesis was applied, using 4-(trifluoromethyl)benzothioamide **1** and ethyl 2-chloroacetoacetate **2**. The key intermediate 2-(4-trifluoromethyl-phenyl)-4-methyl-thiazole-5-carbohydrazide **4** was prepared (yield: 80%) by the reaction of ethyl-2-(4-trifluoromethyl-phenyl)-4-methyl-thiazole-5-carboxylate **3** with hydrazine hydrate in absolute ethanol, according to the literature [26]. The next step was the condensation of compound **4** with differently substituted benzaldehydes, in order to obtain, in good yields (70%–99%), target acyl-hydrazones **5**–**18**.

**Table:** 

Cp	R	R_1_	Cp	R	R_1_
**5**	H	2-OCH_3_	**12**	H	4-Br
**6**	H	3-OCH_3_	**13**	H	4-F
**7**	H	4-OCH_3_	**14**	H	2-OH
**8**	H	3-Cl	**15**	H	3-OH
**9**	H	4-Cl	**16**	H	4-OH
**10**	H	2-Br	**17**	2-Cl	4-Cl
**11**	H	3-Br	**18**	2-Cl	6-Cl

All newly synthesized molecules were purified by recrystallization. Purity was verified by TLC, under UV light exposure. Mass spectrometry confirmed molecular mass of the compounds. ^1^H-NMR spectra showed three characteristic signals for the two methyl and one methylene group, demonstrating the synthesis of ethyl-2-(4-trifluoromethyl-phenyl)-4-methyl-thiazole-5-carboxylate **3**. For thiazole-carbohydrazide **4**, the signals due to NH and NH_2_ protons confirmed the formation of this compound. In the ^1^H-NMR of **5–18**, the NH proton signal shifted from 9.65 ppm in carbohydrazide to 11.84–12.21 ppm region. Also, signals of NH_2_ protons disappeared. The spectra showed characteristic signals for N=CH protons and also for new phenyl protons, which confirmed that condensation with benzaldehyde derivatives took place.

### 2.2. Antimicrobial Screening

Newly synthesized compounds were tested for their antimicrobial activity, using agar diffusion technique. Stock solution of each substance (1 mg/mL) was prepared in dimethylsulfoxide. The screening was done against two gram-negative bacterial strains: *Salmonella enteritidis* ATCC 13,076 and *Escherichia coli* ATCC 25,922, two gram-positive bacterial strains: *Listeria monocytogenes* ATCC 13,932 and *Staphylococcus aureus* ATCC 6538P, and one fungal strain: *Candida albicans* ATCC 10,231. The assessment of the antimicrobial potency was realized by measuring the diameter of the growth inhibition zone. There were two drugs used as reference: gentamicin for the antibacterial activity and fluconazole for the antifungal. DMSO was used as control and it did not present a growth inhibitory effect. The results of the screening are summarized in Table 1.

**Table 1:** Antimicrobial activity of the new synthesized compounds.

Cp (1 mg/mL)	Microbial Strain/Diameter of the Growth Inhibition Zone (mm)				
	Gram-Negative Bacteria		Gram-Positive Bacteria		Fungus
	*S. enteritidis* ATCC 13,076	*E. coli* ATCC 25,922	*L. monocytogenes* ATCC 13,932	*S. aureus* ATCC 6538P	*C. albicans* ATCC 10,231
**3**	20	18	16	12	18
**4**	20	18	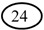	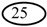	20
**5**	10	16	-	12	25
**6**	10	18	-	12	20
**7**	18	18	14	16	14
**8**	10	18	-	14	20
**9**	18	18	14	14	18
**10**	18	20	-	14	22
**11**	18	20	-	14	20
**12**	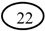	20	14	14	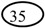
**13**	20	18	14	16	14
**14**	12	16	16	16	18
**15**	18	18	-	12	18
**16**	18	20	-	12	20
**17**	12	20	-	12	18
**18**	18	20	-	12	20
**Gentamicin**	18	22	18	19	NT
**Fluconazole**	NT	NT	NT	NT	25

NT = not tested.

Investigated compounds exerted moderate-to-good activity against microbial strains used. The growth inhibition was more pronounced in case of gram-negative bacteria. The strain of *S. enteritidis* ATCC 13076 was sensitive to all new substances. Seven of the 16 compounds presented the same activity as gentamicin used as reference, while compounds **3**, **4**, **12**, and **13** showed to be more potent than the antibiotic. Analyzing the inhibition of *E. coli* ATCC 25922, six (**10**–**12**, **16**–**18**) of the new thiazole derivatives showed promising potential, the growth inhibition diameters being close to that of gentamicin.

*L. monocytogenes* ATCC 13932 was the less inhibited bacterial strain. It is worth mentioning the activity displayed by aryl-thiazolyl-carbohydrazide **4** was superior to the reference drug. The same compound exerted best inhibition also against *S. aureus* ATCC 6538P, superior to gentamicin.

The antifungal screening revealed that all the investigated derivatives displayed modest-to-good activity. As it can be seen in Table 1, new synthesized molecule **5** showed an inhibitory effect similar to that of fluconazole, used as reference. A very promising candidate is acyl-hydrazone **12**, obtained from condensation with 4-bromo-benzaldehyde, which proved to be the most active, being a more powerful growth inhibitor than the reference.

The good antimicrobial results can be explained by the capacity of acyl-hydrazones to form hydrogen bonds. The presence of aryl ring with various substitutes in different positions attached to the double bond of azomethine group displayed good antifungal activity, according to literature data [27,28]. Also, the most active compounds against fungi species possess electron-withdrawing F, Cl, Br, and CF_3_ at the phenyl ring [29,30]. In our series, 4-Br-phenyl derivative displayed the most powerful growth inhibitory effect against *C. albicans* strain. Para-substitution of phenyl ring was endowed with better antimicrobial properties, this aspect being in agreement with literature [31,32]. These results indicate that introduction of different electron-withdrawing atoms or groups in different positions of the aryl ring [33] plays an important role in the antimicrobial activity of the hydrazones tested here.

### 2.3. DPPH Based Free Radical Scavenging Activity

2,2-Diphenyl-1-picrylhydrazyl (DPPH) radical scavenging is considered a good *in vitro* model, being widely used to conveniently assess antioxidant efficacy, as a rapid, easy, and cost-effective spectrophotometric method [34,35]. DPPH radicals are stable free radicals, which are neutralized in the presence of molecules capable of donating H atoms or electrons. The color of DPPH changes from purple (free radical) to yellow (2,2-diphenyl-1-picrylhydrazine, the non-radicalic, reduced form). This is taken as an indicator of the hydrogen donating ability of the tested compounds. The degree of discoloration indicates the scavenging potential of the antioxidant compounds or samples in terms of hydrogen donating ability. The absorbance is registered at 517 nm.

**Figure 1:**
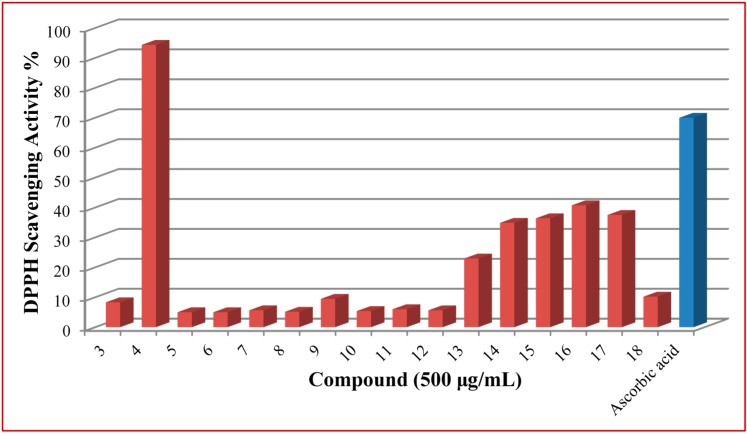
DPPH radical scavenging activity of the new synthesized molecules.

The ability of Schiff bases and their derivatives to scavenge free radicals is an important property. These chemical structures may act as metal chelators of ions involved in lipid oxidation or may react with oxygen bearing systems. The reducing abilities of the examined compounds were determined by their interaction with the free stable radical 2,2-diphenyl-1-picrylhydrazyl (DPPH) at 500 μg/mL concentration, for 30 min. The results of the antioxidant screening are depicted in Figure 1. Ascorbic acid was used as reference.

By comparing structural information and antioxidant activity, it was observed that DPPH radical scavenging antioxidant activity depends on two parameters: the functional groups on the aromatic ring and the position of functional groups on the ring. Antioxidants donate hydrogen atoms to become stable free radicals. The degree of stability and antioxidant potential are in direct relationship with the range of delocalization. All investigated compounds demonstrated an anti-radical activity. For most of the new acyl-hydrazones, the effect was weaker than that of the reference ascorbic acid, but it is worth mentioning the results obtained for compound **4**, 2-(4-trifluoromethyl-phenyl)-4-methyl-thiazol-5-carbohydrazide. In this case, the percentage of inhibition was superior to ascorbic acid. Generally, organic molecules incorporating an electron donating groups, such as amine, hydroxyl, or methoxy, are inquired as promising antioxidant molecules.

Compounds **14**–**16** bearing a hydroxyl group at *orto*, *meta*, or *para* position of the phenyl ring, showed better DPPH activity than the others tested. This result is consistent with the concept that the hydroxyl group enhances antioxidant ability [36,37]. The activities of monohydroxy compounds mainly depend upon the position of the hydroxy group. *Para*-substitution has more stabilizing potential then *meta*-substitution. This can explain why compound **16**, with OH group in position 4 of phenyl ring, displayed better activity than *meta*- and *orto*- derivatives. Lower capacity of compound **14**, with OH group in position 2, may be due to the intramolecular hydrogen bonding.

Derivatives **5**–**7** bearing methoxy group didn’t display a good antioxidant effect. From the results obtained, new substances containing fluorine (**13**), in position *para*, or chlorine (**17**) exhibited better activity than the others. Also, the addition of another chlorine atom on the structure of **9**, in compound **17**, increased the scavenging effect.

The inhibition displayed on the DPPH radical by the test samples shows that the new synthesized hydrazones are capable of neutralizing free radicals and thus, could represent promising candidates for the treatment of pathological diseases and conditions caused as a result of excessive radicals or stress.

## 3. Experimental Section

### 3.1. Reagents and Solvents

Reagents and solvents were obtained from commercial sources: 4-(trifluoromethyl)-benzothioamide from Maybridge, Cornwall, England, UK; ethyl 2-chloroacetoacetate, hydrazine hydrate from Sigma Aldrich, Buchs, Switzerland; substituted benzaldehydes, HPLC grade methanol from Merck, Germany; DPPH (2,2-diphenyl-1-picrylhydrazyl) from Alfa-Aesar (Karlruhe, Germany); DMSO (AppliChem-An ITW Company, Darmstadt, Germany) and used as received. The intermediates were synthesized in our research laboratory.

### 3.2. Analytical Methods

Analytical thin layer chromatography (TLC) was carried out on Merck precoated Silica Gel 60F_254_ sheets (Merck, Darmstadt, Germany), using UV absorption for visualization. Melting points were determined on open glass capillaries with the help of MPM-H1 Schorpp melting point meter (Schorpp Gerätetechnik, Überlingen, Germany), and are uncorrected. The ^1^H-NMR spectra were recorded at room temperature on a Bruker Avance NMR spectrometer (Bruker, Karlsruhe, Germany) operating at 500 MHz and were in accord with the assigned structures. Chemical shift values were reported relative to tetramethylsilane (TMS) as internal standard and are expressed in ppm. The samples were prepared by dissolving the synthesized powder of the compounds in DMSO-*d*_6_ (δ_H_ = 2.51 ppm) as solvent. Mass spectra were recorded by Agilent 1100, type SL spectrometer (positive ionization, Agilent, Santa Clara, CA, USA) and with a Varian MAT CH-5 spectrometer (70 eV, Varian MAT, Bremen, Germany). Elemental analysis was registered with a Vario El CHNS instrument (Elementar Analysensysteme GmbH, Hanau, Germany).

### 3.3. Chemical Synthesis

#### 3.3.1. General Procedure for the Synthesis of Ethyl-4-methyl-2-(4-(trifluoromethyl)phenyl)thiazole-5-carboxylate (**3**)

To a solution of 4-(trifluoromethyl)benzothioamide **1** (10 mmol/2.05 g) in the minimum required quantity of absolute ethanol (10 mL), ethyl 2-chloroacetoacetate **2** (15 mmol/2.46 g) was added and the resulting mixture was heated on the water bath for 5 h. The solution was cooled down and neutralized with a solution of potassium carbonate. The resulting solid was filtered, washed with water, recrystallized from ethanol to give the target compound.

*Ethyl-4-methyl-2-(4-(trifluoromethyl)phenyl)thiazole-5-carboxylate* (**3**): light brown powder, yield: 80%, mp: 90 °C. ^1^H-NMR (DMSO-*d*_6_, 500 MHz, ppm): δ 1.30 (t, 3H, -CH_3_); 2.68 (s, 3H, -CH_3_); 4.30 (q, 2H, -CH_2_-); 7.83 (d, 2H, phenyl-H); 8.15 (d, 2H, phenyl-H). Anal. Calcd. (%) for C_14_H_12_F_3_NO_2_S (315.31): C, 53.33; H, 3.84; N, 4.44; S, 10.17. Found: C, 53.32; H, 3.84; N, 4.43; S, 10.18. MS (EI, 70 eV): *m*/*z* (%) 316 [M^+^], 288 (100%).

#### 3.3.2. General Procedure for the Synthesis of 4-Methyl-2-(4-(trifluoromethyl)phenyl)thiazole-5-carbohydrazide (**4**)

To a solution of compound **3** (10 mmol/3.15 g) in absolute ethanol (20 mL), hydrazine hydrate (15 mmol/0.75 g) was added and the resulting mixture was heated on water bath, for 5 h. The mixture was filtered while still hot and the obtained solution was cooled down. A yellow precipitate was formed, washed with water and recrystallized from ethanol, in order to obtain carbohydrazide **4**.

*4-Methyl-2-(4-(trifluoromethyl)phenyl)thiazole-5-carbohydrazide* (**4**): white-light green powder, yield: 80%, mp: 177 °C. ^1^H-NMR (DMSO-*d*_6_, 500 MHz, ppm): δ 2.63 (s, 3H, -CH_3_); 4.60 (br, 2H, -NH_2_); 7.87 (d, 2H, phenyl-H); 8.13 (d, 2H, phenyl-H); 9.65 (br, 1H, -NH-). Anal. Calcd. (%) for C_12_H_10_F_3_N_3_OS (301.29): C, 47.84; H, 3.35; N, 13.95; S, 10.64. Found: C, 47.83; H, 3.35; N, 13.94; S, 10.65. MS (EI, 70 eV): *m*/*z* (%) 302 [M^+^], 285 (25%), 244 (100%).

#### 3.3.3. General Procedure for the Synthesis of Acyl-hydrazones **5**–**18**

To a solution of thiazole-carbohydrazide **4** (1 mmol/0.301 g) in absolute ethanol (10 mL), 1 mmol of different substituted benzaldehyde and two drops of concentrated sulphuric acid were added. The mixture was heated for 4 h. The residue was filtered and recrystallized from ethanol in order to give compounds **5**–**18**, as different color powders.

*N*′*-(2-Methoxybenzylidene)-4-methyl-2-(4-(trifluoromethyl)phenyl)thiazole-5-carbohydrazide* (**5**): yellow powder, yield: 98%, mp: 289 °C. ^1^H-NMR (DMSO-*d*_6_, 500 MHz, ppm): δ 2.80 (s, 3H, -CH_3_); 3.87 (s, 3H, -CH_3_); 7.10–7.15 (m, 2H, phenyl-H); 7.46 (t, 1H, phenyl-H); 7.92 (d, 1H, phenyl-H); 7.95 (d, 2H, phenyl-H); 8.23 (d, 2H, phenyl-H); 8.49 (s, 1H, =CH-); 11.94 (s, 1H, -NH-). Anal. Calcd. (%) for C_20_H_16_F_3_N_3_O_2_S (419.42): C, 57.27; H, 3.85; N, 10.02; S, 7.65. Found: C, 57.26; H, 3.84; N, 10.01; S, 7.66. MS (EI, 70 eV): *m*/*z* (%) 420 [M^+^], 270 (25%), 244 (100%).

*N′-(3-Methoxybenzylidene)-4-methyl-2-(4-(trifluoromethyl)phenyl)thiazole-5-carbohydrazide* (**6**): light yellow powder, yield: 90%, mp: 249 °C. ^1^H-NMR (DMSO-*d*_6_, 500 MHz, ppm): δ 2.81 (s, 3H, -CH_3_); 3.85 (s, 3H, -CH_3_); 7.03–7.05 (dd, 1H, phenyl-H); 7.33 (d, 1H, phenyl-H); 7.41 (d, 1H, phenyl-H); 7.44 (s, 1H, phenyl-H); 7.92 (d, 2H, phenyl-H); 8.12 (s, 1H, =CH-); 8.22 (d, 2H, phenyl-H); 12.02 (s, 1H, -NH-). Anal. Calcd. (%) for C_20_H_16_F_3_N_3_O_2_S (419.42): C, 57.27; H, 3.85; N, 10.02; S, 7.65. Found: C, 57.26; H, 3.85; N, 10.01; S, 7.66. MS (EI, 70 eV): *m*/*z* (%) 420 [M^+^], 270 (7%), 244 (100%).

*N′-(4-Methoxybenzylidene)-4-methyl-2-(4-(trifluoromethyl)phenyl)thiazole-5-carbohydrazide* (**7**): yellow powder, yield: 87%, mp: 232 °C. ^1^H-NMR (DMSO-*d*_6_, 500 MHz, ppm): δ 2.80 (s, 3H, -CH_3_); 3.82 (s, 3H, -CH_3_); 7.08 (d, 2H, phenyl-H); 7.74 (d, 2H, phenyl-H); 7.92 (d, 2H, phenyl-H); 8.09 (s, 1H, =CH-); 8.25 (d, 2H, phenyl-H); 11.84 (s, 1H, -NH-). Anal. Calcd. (%) for C_20_H_16_F_3_N_3_O_2_S (419.42): C, 57.27; H, 3.85; N, 10.02; S, 7.65. Found: C, 57.26; H, 3.85; N, 10.02; S, 7.66. MS (EI, 70 eV): *m/z* (%) 420 [M^+^], 244 (100%).

*N′-(3-Chlorobenzylidene)-4-methyl-2-(4-(trifluoromethyl)phenyl)thiazole-5-carbohydrazide* (**8**): light yellow powder, yield: 99%, mp: 263 °C. ^1^H-NMR (DMSO-*d*_6_, 500 MHz, ppm): δ 2.81 (s, 3H, -CH_3_); 7.03–7.04 (dd, 1H, phenyl-H); 7.31 (d, 1H, phenyl-H); 7.43 (d, 1H, phenyl-H); 7.45 (s, 1H, phenyl-H); 7.92 (d, 2H, phenyl-H); 8.10 (s, 1H, =CH-); 8.21 (d, 2H, phenyl-H); 12.00 (s, 1H, -NH-). Anal. Calcd. (%) for C_19_H_13_ClF_3_N_3_OS (423.84): C, 53.84; H, 3.09; N, 9.91; S, 7.57. Found: C, 53.85; H, 3.08; N, 9.92; S, 7.56. MS (EI, 70 eV): *m*/*z* (%) 424 [M^+^], 244 (100%).

*N′-(4-Chlorobenzylidene)-4-methyl-2-(4-(trifluoromethyl)phenyl)thiazole-5-carbohydrazide* (**9**): yellow powder, yield: 86%, mp: 290 °C. ^1^H-NMR (DMSO-*d*_6_, 500 MHz, ppm): δ 2.78 (s, 3H, -CH_3_); 7.08 (d, 2H, phenyl-H); 7.72 (d, 2H, phenyl-H); 7.92 (d, 2H, phenyl-H); 8.07 (s, 1H, =CH-); 8.20 (d, 2H, phenyl-H); 11.87 (s, 1H, -NH-). Anal. Calcd. (%) for C_19_H_13_ClF_3_N_3_OS (423.84): C, 53.84; H, 3.09; N, 9.91; S, 7.57. Found: C, 53.84; H, 3.08; N, 9.92; S, 7.56. MS (EI, 70 eV): *m*/*z* (%) 424 [M^+^], 244 (100%).

*N′-(2-Bromobenzylidene)-4-methyl-2-(4-(trifluoromethyl)phenyl)thiazole-5-carbohydrazide* (**10**): light yellow powder, yield: 98%, mp: 289 °C. ^1^H-NMR (DMSO-*d*_6_, 500 MHz, ppm): δ 2.80 (s, 3H, -CH_3_); 7.10–7.14 (m, 2H, phenyl-H); 7.41 (t, 1H, phenyl-H); 7.94 (d, 1H, phenyl-H); 7.96 (d, 2H, phenyl-H); 8.20 (d, 2H, phenyl-H); 8.41 (s, 1H, =CH-); 11.89 (s, 1H, -NH-). Anal. Calcd. (%) for C_19_H_13_BrF_3_N_3_OS (468.29): C, 48.73; H, 2.80; N, 8.97; S, 6.85. Found: C, 48.74; H, 2.80; N, 8.98; S, 6.86. MS (EI, 70 eV): *m*/*z* (%) 468 [M^+^], 451 (6%), 244 (100%).

*N′-(3-Bromobenzylidene)-4-methyl-2-(4-(trifluoromethyl)phenyl)thiazole-5-carbohydrazide* (**11**): light yellow powder, yield: 89%, mp: 279 °C. ^1^H-NMR (DMSO-*d*_6_, 500 MHz, ppm): δ 2.78 (s, 3H, -CH_3_); 7.03–7.06 (dd, 1H, phenyl-H); 7.37 (d, 1H, phenyl-H); 7.45 (d, 1H, phenyl-H); 7.51 (s, 1H, phenyl-H); 7.90 (d, 2H, phenyl-H); 8.09 (s, 1H, =CH-); 8.23 (d, 2H, phenyl-H); 11.97 (s, 1H, -NH-). Anal. Calcd. (%) for C_19_H_13_BrF_3_N_3_OS (468.29): C, 48.73; H, 2.80; N, 8.97; S, 6.85. Found: C, 48.74; H, 2.80; N, 8.97; S, 6.86. MS (EI, 70 eV): *m*/*z* (%) 468 [M^+^], 451 (6%), 244 (100%).

*N′-(4-Bromobenzylidene)-4-methyl-2-(4-(trifluoromethyl)phenyl)thiazole-5-carbohydrazide* (**12**): yellow powder, yield: 91%, mp: 296 °C. ^1^H-NMR (DMSO-*d*_6_, 500 MHz, ppm): δ 2.74 (s, 3H, -CH_3_); 7.06 (d, 2H, phenyl-H); 7.77 (d, 2H, phenyl-H); 7.91 (d, 2H, phenyl-H); 8.09 (s, 1H, =CH-); 8.23 (d, 2H, phenyl-H); 11.91 (s, 1H, -NH-). Anal. Calcd. (%) for C_19_H_13_BrF_3_N_3_OS (468.29): C, 48.73; H, 2.80; N, 8.97; S, 6.85. Found: C, 48.73; H, 2.80; N, 8.96; S, 6.86. MS (EI, 70 eV): *m*/*z* (%) 468 [M^+^], 451 (6%), 244 (100%).

*N′-(4-Fluorobenzylidene)-4-methyl-2-(4-(trifluoromethyl)phenyl)thiazole-5-carbohydrazide* (**13**): yellow powder, yield: 71%, mp: 304 °C. ^1^H-NMR (DMSO-*d*_6_, 500 MHz, ppm): δ 2.79 (s, 3H, -CH_3_); 7.07 (d, 2H, phenyl-H); 7.74 (d, 2H, phenyl-H); 7.94 (d, 2H, phenyl-H); 8.10 (s, 1H, =CH-); 8.25 (d, 2H, phenyl-H); 11.98 (s, 1H, -NH-). Anal. Calcd. (%) for C_19_H_13_F_4_N_3_OS (407.38): C, 56.02; H, 3.22; N, 10.31; S, 7.87. Found: C, 56.01; H, 3.22; N, 10.30; S, 7.88. MS (EI, 70 eV): *m*/*z* (%) 407 [M^+^], 244 (100%).

*N′-(2-Hydroxybenzylidene)-4-methyl-2-(4-(trifluoromethyl)phenyl)thiazole-5-carbohydrazide* (**14**): yellow powder, yield: 70%, mp: 217 °C. ^1^H-NMR (DMSO-*d*_6_, 500 MHz, ppm): δ 2.79 (s, 3H, -CH_3_); 6.92–7.00 (m, 2H, phenyl-H); 7.28–7.33 (m, 2H, phenyl-H); 7.41 (t, 1H, phenyl-H); 7.92 (d, 1H, phenyl-H); 8.22 (d, 2H, phenyl-H); 8.47 (s, 1H, =CH-); 9.01 (s, 1H, phenyl-OH); 11.90 (s, 1H, -NH-). Anal. Calcd. (%) for C_19_H_14_F_3_N_3_O_2_S (405.39): C, 56.29; H, 3.48; N, 10.37; S, 7.91. Found: C, 56.28; H, 3.48; N, 10.36; S, 7.92. MS (EI, 70 eV): *m*/*z* (%) 406 [M^+^], 270 (24%), 244 (100%).

*N′-(3-Hydroxybenzylidene)-4-methyl-2-(4-(trifluoromethyl)phenyl)thiazole-5-carbohydrazide* (**15**): light yellow powder, yield: 72%, mp: 270 °C. ^1^H-NMR (DMSO-*d*_6_, 500 MHz, ppm): δ 2.76 (s, 3H, -CH_3_); 7.03–7.05 (dd, 1H, phenyl-H); 7.36 (d, 1H, phenyl-H); 7.46 (d, 1H, phenyl-H); 7.53 (s, 1H, phenyl-H); 7.92 (d, 2H, phenyl-H); 8.10 (s, 1H, =CH-); 8.25 (d, 2H, phenyl-H); 9.00 (s, 1H, phenyl-OH); 12.00 (s, 1H, -NH-). Anal. Calcd. (%) for C_19_H_14_F_3_N_3_O_2_S (405.39): C, 56.29; H, 3.48; N, 10.37; S, 7.91. Found: C, 56.28; H, 3.48; N, 10.36; S, 7.92. MS (EI, 70 eV): *m*/*z* (%) 406 [M^+^], 244 (100%).

*N′-(4-Hydroxybenzylidene)-4-methyl-2-(4-(trifluoromethyl)phenyl)thiazole-5-carbohydrazide* (**16**): light yellow powder, yield: 95%, mp: 284 °C. ^1^H-NMR (DMSO-*d*_6_, 500 MHz, ppm): δ 2.78 (s, 3H, -CH_3_); 7.04 (d, 2H, phenyl-H); 7.78 (d, 2H, phenyl-H); 7.91 (d, 2H, phenyl-H); 8.09 (s, 1H, =CH-); 8.23 (d, 2H, phenyl-H); 9.04 (s, 1H, phenyl-OH); 11.96 (s, 1H, -NH-). Anal. Calcd. (%) for C_19_H_14_F_3_N_3_O_2_S (405.39): C, 56.29; H, 3.48; N, 10.37; S, 7.91. Found: C, 56.28; H, 3.48; N, 10.36; S, 7.92. MS (EI, 70 eV): *m*/*z* (%) 406 [M^+^], 244 (100%).

*N′-(2,4-Dichlorobenzylidene)-4-methyl-2-(4-(trifluoromethyl)phenyl)thiazole-5-carbohydrazide* (**17**): light yellow powder, yield: 99%, mp: 298 °C. ^1^H-NMR (DMSO-*d*_6_, 500 MHz, ppm): δ 2.80 (s, 3H, -CH_3_); 7.77 (d, 1H, phenyl-H); 7.91 (d, 2H, phenyl-H); 8.09 (d, 1H, phenyl-H); 8.14 (s, 1H, phenyl-H); 8.25 (d, 2H, phenyl-H); 8.50 (s, 1H, =CH-); 12.21 (br, 1H, -NH-). Anal. Calcd. (%) for C_19_H_12_Cl_2_F_3_N_3_OS (458.28): C, 49.80; H, 2.64; N, 9.17; S, 7.00. Found: C, 49.80; H, 2.64; N, 9.16; S, 7.01. MS (EI, 70 eV): *m*/*z* (%) 459 [M^+^], 287 (9%), 244 (100%).

*N′-(2,6-Dichlorobenzylidene)-4-methyl-2-(4-(trifluoromethyl)phenyl)thiazole-5-carbohydrazide* (**18**): light yellow powder, yield: 94%, mp: 268 °C. ^1^H-NMR (DMSO-*d*_6_, 500 MHz, ppm): δ 2.77 (s, 3H, -CH_3_); 7.48 (t, 1H, phenyl-H); 7.60 (d, 2H, phenyl-H); 7.89 (d, 2H, phenyl-H); 8.12 (s, 1H, phenyl-H); 8.35 (d, 1H, phenyl-H); 8.50 (s, 1H, =CH-); 12.20 (br, 1H, -NH-). Anal. Calcd. (%) for C_19_H_12_Cl_2_F_3_N_3_OS (458.28): C, 49.80; H, 2.64; N, 9.17; S, 7.00. Found: C, 49.79; H, 2.64; N, 9.16; S, 7.01. MS (EI, 70 eV): *m*/*z* (%) 459 [M^+^], 287 (9%), 244 (100%).

### 3.4. Antimicrobial Screening

The screening of antimicrobial activity was done in the State Veterinary Laboratory for Animal Health and Safety, Cluj-Napoca, Romania, according to the guidelines of National Committee for Clinical Laboratory Standards (NCCLS, 1997), [38], using the agar diffusion method. Gentamicin and fluconazole were purchased from the drug market and used as reference for antibacterial and antifungal activity, respectively. Petri plates containing 20 mL of Mueller Hinton Agar were used for all the bacteria tested and Mueller-Hinton medium supplemented with 2% glucose (providing adequate growth of yeasts) and 0.5 g/L methylene blue (providing a better definition of the inhibition zone diameter) was used for antifungal testing.

After 18 h, the bacterial strains were put on a saline solution of NaCl (0.9%), so that the turbidity would be that of MacFarland (10^6^ UFC/mL). The inoculum was spread on the surface of the solidified media. Solutions of the tested compounds were prepared in DMSO and concentration of 1 mg/mL was used.

Six-millimeter diameter wells were cut from the agar using a sterile cork-borer. A sterile swab was soaked in suspension and then the Mueller-Hinton agar plates were inoculated by streaking the entire surface. After drying for 10–15 min, the six millimeter diameter wells were inoculated with 50 μL from each solution. Gentamicin (10 μg/well) and fluconazole (25 μg/well) were used as antibacterial and antifungal reference, respectively. Plates inoculated with bacteria were incubated for 24 h and those with fungus 48 h, at 37 °C.

The effects of the new compounds were assessed by measuring the diameter of the growth inhibition zone. Zone diameters were measured to the nearest whole millimeter at a point in which there will be no visible growth after 24–48 h.

All the tests were performed in duplicate and the average was taken as final reading. All microorganism products were distributed by MicroBioLogics^®^: *Salmonella enteritidis* ATCC 13076 (Gram−), *Escherichia coli* ATCC 25922 (Gram−), *Listeria monocytogenes* ATCC 13932 (Gram+), *Staphylococcus aureus* ATCC 6538P (Gram+), and *Candida albicans* ATCC 10231 (fungus).

### 3.5. DPPH Based Free Radical Scavenging Activity

The direct antioxidant activity of the tested compounds was evaluated through a free radical scavenging assay, namely the stable DPPH radical method. Samples were prepared by treating a series of methanolic 0.1 g/L DPPH solutions with an equal volume of the tested thiazole hydrazones solution, of 1 mg/mL concentration. Simultaneously, a control sample was prepared by diluting 1:1 the initial DPPH solution with methanol. The mixtures where incubated for 30 min at 40 °C and then the absorbance was measured at 517 nm. A decrease in absorbance is associated with the reduction of the DPPH radical and thus directly proportional to the radical scavenging activity of the tested compounds. The DPPH scavenging ability was expressed as a percentage of absorbance reduction: DPPH scavenging ability % = [(A_control_ − A_sample_)/A_control_] × 100, where Abs_control_ is the absorbance of DPPH radical + methanol (containing all reagents except the sample) and Abs_sample_ is the absorbance of DPPH radical + compound sample. As a positive control we used the well-known antioxidant, ascorbic acid.

The investigation of the antioxidant potential was completed in the research laboratory of Pharmacognosy Department of Faculty of Pharmacy, Cluj-Napoca, Romania.

## 4. Conclusions

We have successfully synthesized new hydrazones, derivatives of 2-(4-trifluoromethyl-phenyl)-4-methyl-thiazol-5-carbohydrazide. The compounds were isolated in solid state, in high yields (70%–99%), characterized with the help of combined physico-chemical and spectral (MS, ^1^H-NMR) data. The antimicrobial effect of the new molecules was investigated using the diffusion technique. 5-Thiazole-carbohydrazide **4** showed a strong growth inhibitory activity, higher than that of gentamicin, the reference antibiotic used, against Gram-positive strains and against the strain of *S. enteritidis*. Compound **12**, a 4-bromo-phenyl derivative, displayed a good inhibitory effect against *S. enteritidis*, too. This new molecule represents a very promising candidate for the antifungal activity, the effect being superior to that of fluconazole, the antimycotic reference, on *C. albicans*. All compounds manifested an anti-radical potential. The best DPPH scavenging activity was demonstrated by carbohydrazide **4**, better than ascorbic acid. The obtained results suggest that the new hydrazones bearing thiazole scaffold may be considered for further investigation and optimization, in designing antimicrobial and antioxidant drugs.
